# Bouveret Syndrome—The Rarest Variant of Gallstone Ileus: A Case Report and Literature Review

**DOI:** 10.1155/2013/839370

**Published:** 2013-06-24

**Authors:** Vasileios K. Mavroeidis, Dimitrios I. Matthioudakis, Nikolaos K. Economou, Ioannis D. Karanikas

**Affiliations:** 1st Surgical Department, Sismanogleio General Hospital of Attica, 15126 Athens, Greece

## Abstract

We present a case report of a patient with Bouveret syndrome with interesting radiological findings and successful surgical treatment after failure of the endoscopic techniques. The report is followed by a review of the literature regarding the diagnostic means and proper treatment of this rare entity. Bouveret syndrome refers to the condition of gastric outlet obstruction caused by the impaction of a large gallstone into the duodenum after passage through a cholecystoduodenal fistula. Many endoscopic and surgical techniques have been described in the management of this syndrome. This is a case of a 78-year-old patient with severe medical history who presented in bad general condition with an 8-day history of nausea, multiple bilious vomiting episodes, anorexia, discomfort in the right hypochondrium and epigastrium, and fever up to 38,5°C. The diagnosis of Bouveret syndrome was set after performing the proper imaging studies. An initial endoscopic effort to resolve the obstruction was performed without success. Surgical treatment managed to extract the impacted gallstone through an enterotomy after removal into the first part of the jejunum.

## 1. Introduction

Bouveret syndrome is a very uncommon form of gallstone ileus caused by the passage and impaction of a large gallstone through a cholecystoduodenal fistula into the duodenum, resulting in gastric outlet obstruction. It was first described in 1770 by Beaussier [1, 2]. In 1896 the French physician Leon Bouveret published two cases of gastric outlet obstruction due to gallstone impaction in the duodenal bulb [1, 2]. Gallstone ileus is responsible for 1%–4% of all cases with small-bowel obstruction [3]. The most common location of a calculus causing obstruction is the terminal ileum [1–5]. More proximal impaction is unusual whereas obstruction of the duodenum accounts for 1%–3% of all cases [2, 4, 6].

Until 2008, up to 300 cases had been reported in the world literature [4]. Morbidity and mortality rates have decreased in recent years but still remain high, estimated at 60% and 12%–30% respectively, due to the advanced age and the comorbid factors of the patients [4–7]. These rates strictly depend on the therapeutic approach [4]. A variety of endoscopic and surgical techniques have been described in the treatment of Bouveret syndrome.

 We present a case of Bouveret syndrome with interesting radiological findings and successful surgical management following endoscopic treatment failure, followed by a review of the literature.

## 2. Case Report

 A 78-year-old Caucasian (Greek origin) female patient presented with an 8-day history of nausea, multiple bilious vomiting episodes, anorexia, discomfort in the right hypochondrium and epigastrium and fever up to 38,5°C.

 On physical examination the patient was in bad condition with affected facet, pale skin and mucosae, signs of dehydration, tachypnoea, tachyarrhythmia and fever (39°C), while the abdomen was soft, nontender, mildly distended and painful on palpation of the epigastrium and right upper quadrant. 

 Her medical history included a HCV infection, arterial hypertension, chronic atrial fibrillation, diabetes mellitus type 2, stroke with residual left hemiparesis, hypothyroidism, hyperlipidaemia and consumption of NSAIDs over the previous week.

 Laboratory examinations revealed a white blood cell count of 10.72 (×10^9^/L), Neu 88.5%, toxic granulation of polymorphonuclears, a C-reactive protein of 72.7 (IU/L), Glu 139 (IU/L), Urea 73.4 (IU/L), Crea 1.38 (IU/L), *γ*GT 87 (IU/L), tBIL 1.37 (IU/L), dBIL 1.10 (IU/L), and LDH 517 (IU/L).

**Figure 1:**
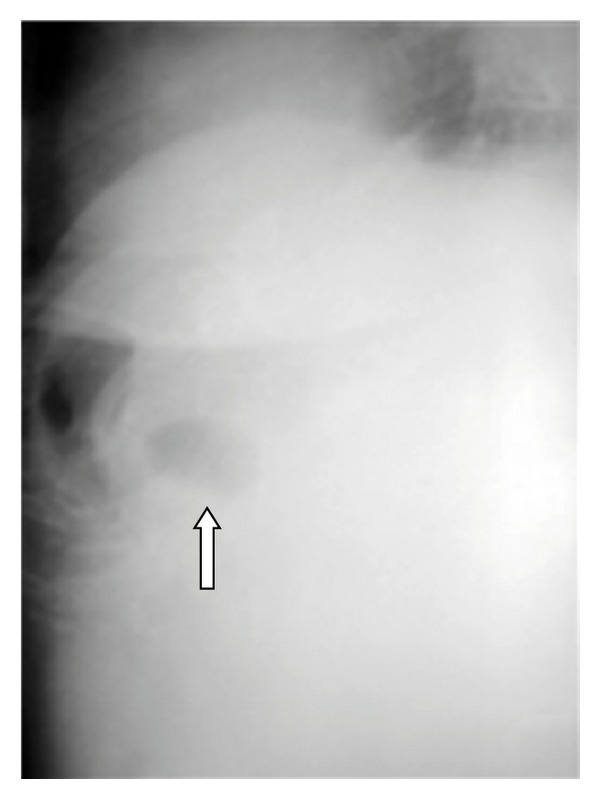
A plain abdominal X-ray suspecting the presence of air in the gallbladder (arrow).

 The chest X-ray revealed a possible aspirational pneumonia. The abdominal X-ray was suspicious for presence of air in the gallbladder (Figure 1).

On abdominal U/S the gallbladder could not be well visualised, the presence of air in the intrahepatic bile ducts was suspected and no dilatation of the bile ducts was detected. The stomach was found dilated and filled with liquid.

**Figure 2:**
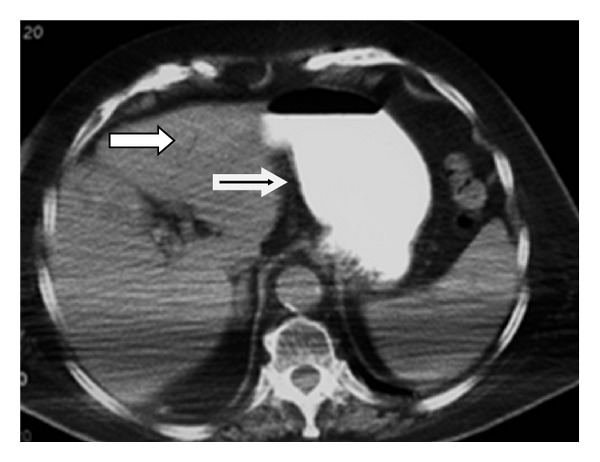
Small arrow showing the presence of air in an intrahepatic bile duct. Large arrow showing gastric dilatation.

**Figure 3:**
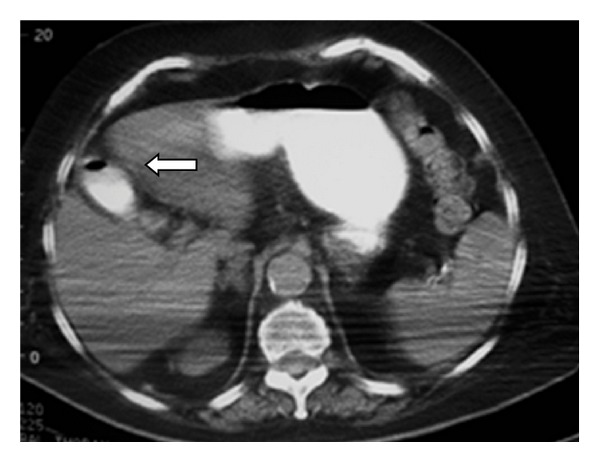
Contrast and air inside the gallbladder (arrow).

**Figure 4:**
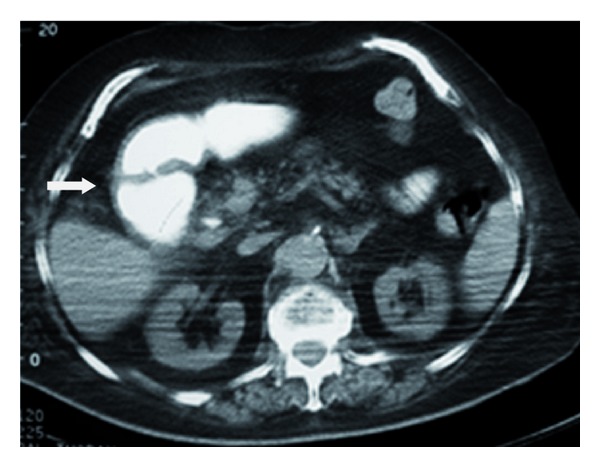
Dilatation of the duodenum (arrow).

**Figure 5:**
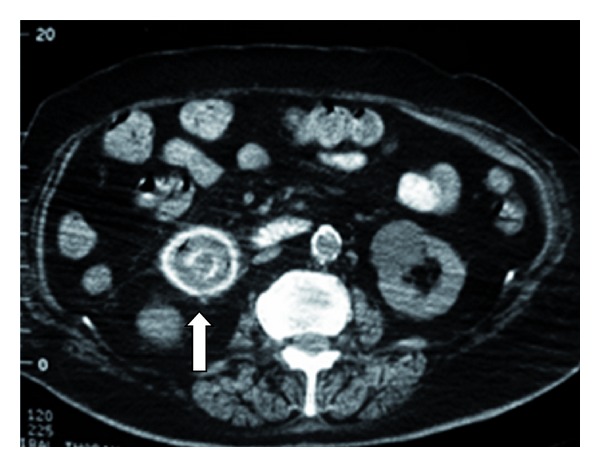
A gallstone in the 2nd to 3rd part of the duodenum (arrow).

Abdominal CT showed the presence of a gallstone in the 2nd to 3rd part of the duodenum and a dilatation of the first part. The presence of contrast and air in the gallbladder was also noticed. The stomach was found with mild dilatation (Figures 2, 3, 4, and 5).

The diagnosis “Bouveret syndrome” was set. A nasogastric tube was placed to decompress the stomach. Initial treatment included administration of fluids, electrolytes and antibiotics.

**Figure 6:**
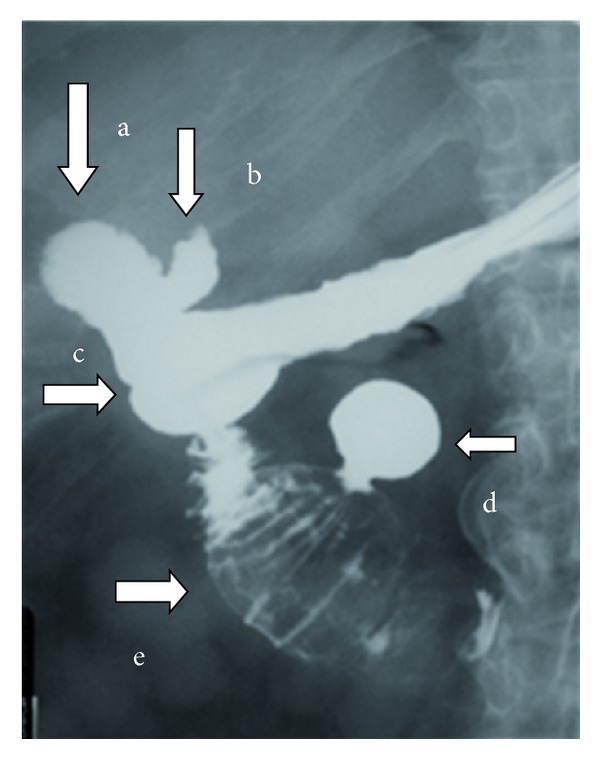
Five minutes after intake of the oral contrast all the anatomic elements are well visualised: (a) the gallbladder filled with contrast, (b) the cystic duct filled with contrast, (c) the cholecystoduodenal fistula, (d) a duodenal diverticulum of the 3rd part filled with contrast, and (e) a large gallstone impacted on the 2nd to 3rd part of the duodenum.

**Figure 7:**
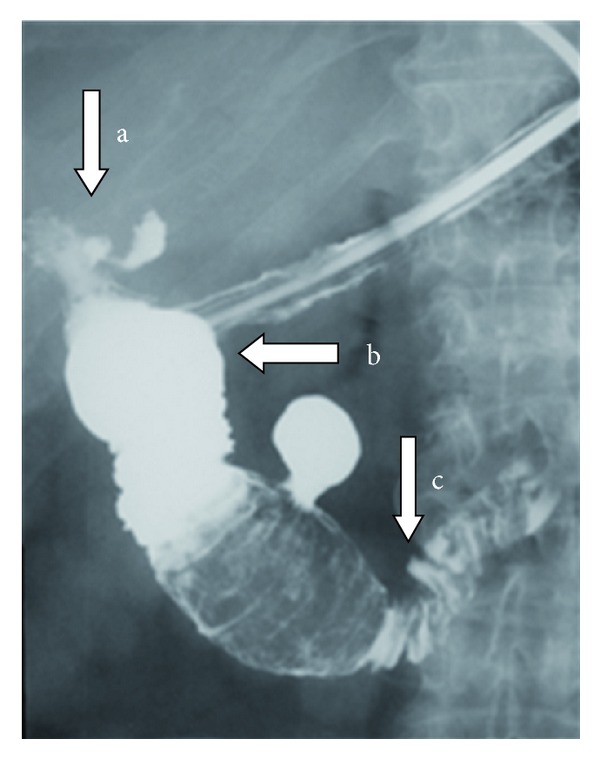
Fifteen minutes after intake of the oral contrast (a) the evacuation of the gallbladder is well seen as well as (b) the dilatation of the filled up with contrast duodenum proximally to the impacted gallstone and (c) the slight passage of the gastrografin distally to the gallstone in the 4rth part and jejunum.

 For further topographic information a gastrografin meal was performed. A large gallstone was visualised in the 2nd to 3rd part of the duodenum. The gallbladder, the cystic duct and the cholecystoduodenal fistula as well as a duodenal diverticulum of the 3rd part were well visualised (Figures 6 and 7).

**Figure 8:**
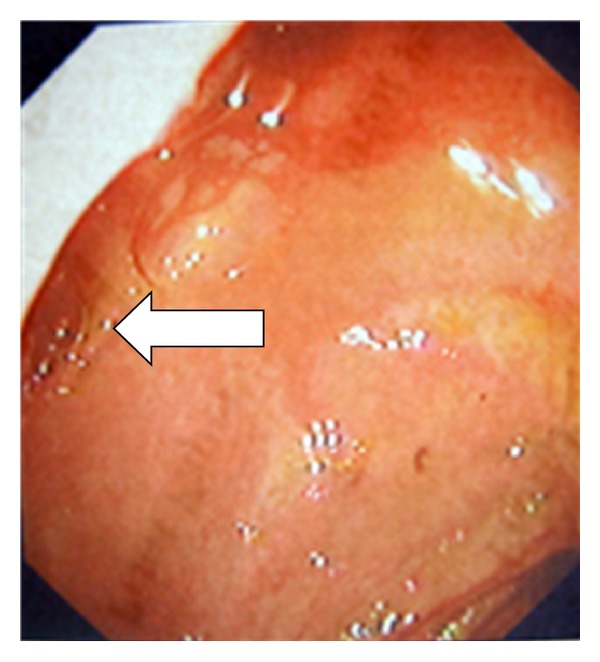
A cholecystoduodenal fistula orifice noticed in the superior duodenal wall (arrow).

**Figure 9:**
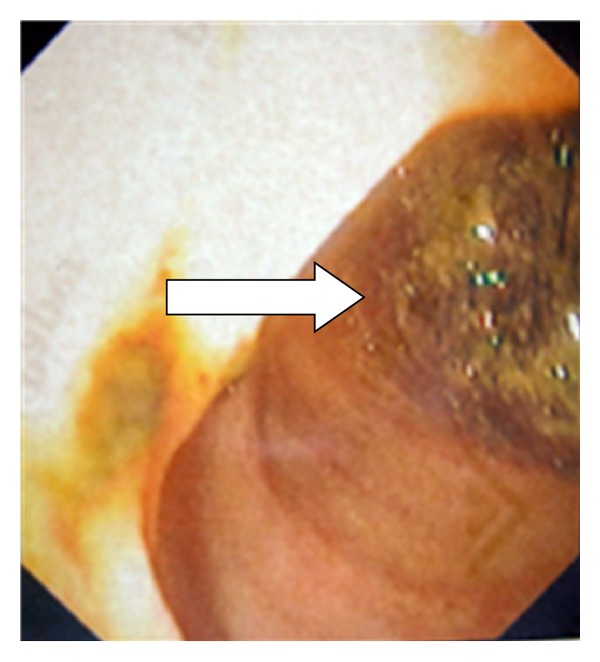
The proximal side of a large gallstone impacted in the 2nd to 3rd part of the duodenum (arrow).

The patient was transferred in the endoscopic unit where a gastroduodenoscopy was performed. The fistula orifice was seen (Figure 8) as well as the proximal side of the gallstone (Figure 9). 

**Figure 10:**
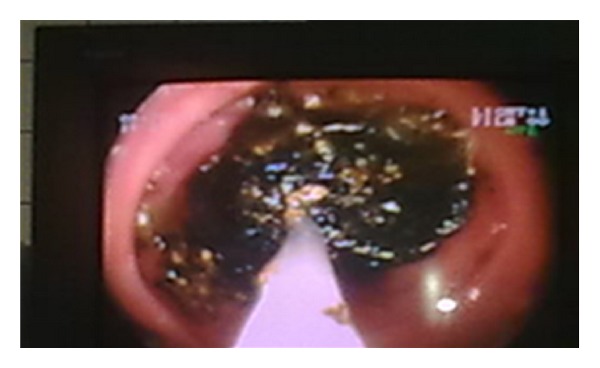


Endoscopic removal and mechanical lithotripsy were attempted using different skills and equipment, but all efforts failed (Figure 10). 

**Figure 11:**
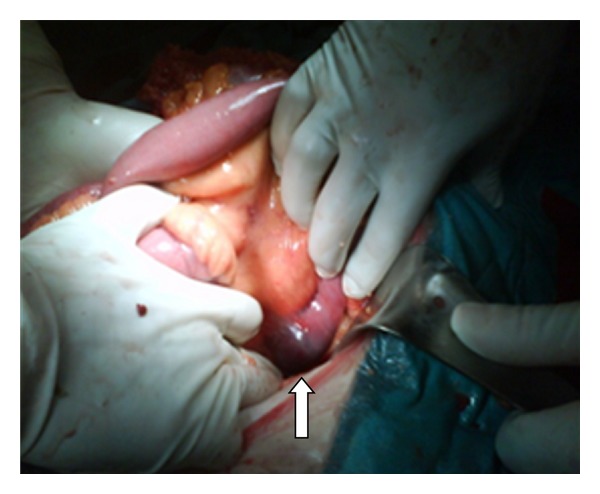
The gallstone located in the first part of the jejunum after successful milking right before jejunotomy (arrow).

**Figure 12:**
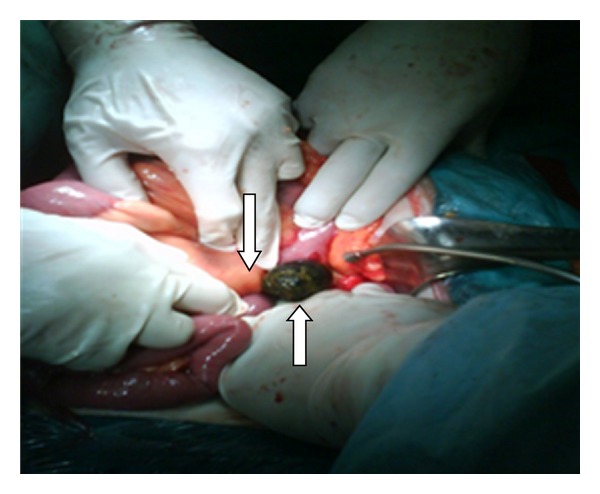
The extraction of the gallstone right after the ligament of Treitz (arrows).

Surgical treatment was decided. Due to serious comorbidity the patient received high thoracic epidural anaesthesia. A laparotomy with midline incision was performed. The gallbladder was found collapsed and the cholecystoduodenal fistula was identified. A Kocher's manoeuvre was performed and the gallstone was palpated in the 2nd to 3rd part of the duodenum. An initial effort for proximal removal towards the stomach was unsuccessful. With gentle milking movements distal removal was achieved in the first part of the jejunum right after the ligament of Treitz (Figure 11). No further removal was feasible. A jejunotomy and successful removal of the gallstone were performed (Figure 12). The size of the extracted calculus was 5.8 × 3.7 × 4 cm. The jejunotomy was sutured in two layers. The gallbladder and the fistula were left intact. No other stones were detected in the digestive tract. No complications occurred after surgery and the patient was discharged on the 8th postoperative day. 

## 3. Discussion

 Biliodigestive fistulae are a very rare complication of cholelithiasis occurring in less than 1% of all patients [4, 5]. In 60% (53–68%) of the cases the fistula is cholecystoduodenal [1, 4, 5]. Less common variants are cholecystocolic in 17%, cholecystogastric in 5% and choledochoduodenal in 5% of the cases [4, 5]. The formation of a fistula is favoured by the long history of cholelithiasis, the repeated episodes of acute cholecystitis, the large size of the calculi (2–8 cm), the female gender and the advanced age of the patients (more than 60 years) [4]. 

 The passage of a large gallstone through the fistula may lead to different manifestations depending on its size, the part of the alimentary tract involved in the fistula and the pre-existence of stenotic areas at this level [4]. In 85% of all cases the gallstone is eliminated either by vomiting or with faeces. On the contrary, 15% of the patients will present in with gallstone ileus due to the impaction of the stone at a different level of the digestive tract [4, 6]. It is estimated that gallstone ileus occurs in 0.3–0.4% of all patients with cholelithiasis and it is seven times more frequent in patients aged over 70 [1]. The term classic or typical gallstone ileus usually refers to the obstruction of the terminal ileum from a calculus [1, 6, 7]. That is the most common location of the obstructing gallstone accounting for 50–90% of all cases [1, 2, 4]. The size of a stone obstructing the terminal ileum is usually more than 2.5 cm [2]. Less common locations are the proximal ileum and the jejunum (20–40%), the colon (3–25%) and more rarely the duodenum or the stomach (1–10%) [1, 2, 4, 6]. Bouveret syndrome refers to the situation occurring by the proximal impaction of a biliary calculus into the duodenum or the distal stomach resulting in gastric outlet obstruction in most cases. Gallstones obstructing the duodenum are usually more than 2.5 cm in size [3].

 Bouveret syndrome is extremely rare and most commonly affects elderly women with a mean age of 68.6 years [1, 2, 6, 7]. More than 60% of the reported cases have an associated previous clinical history [1] while the majority of patients have known biliary lithiasis [2]. The clinical presentation can be different and nonspecific. Usually, the symptoms begin 5 to 7 days before the medical consultation [1]. It has been reported that 43–68% of patients have a history of recent biliary colic bouts, jaundice or acute cholecystitis, but the first manifestation of cholelithiasis as Bouveret syndrome is possible as well [1]. Nausea, vomiting and abdominal pain in the epigastrium and right hypochondrium are frequent [2, 6, 7], but their intensity often does not correlate with the underlying anatomic alterations [1]. Fever, signs of dehydration and weight loss can be present as well [2]. Sepsis is an unusual manifestation [1]. Less frequently, Bouveret syndrome may present with haematemesis [1, 5–7] secondary to duodenal [1, 6] or celiac artery erosions [6]. The expulsion of a stone during vomiting or the presence of oesophageal lesions due to intense vomiting are also possible [1]. Given the clinical characteristics which are mainly nonspecific as well as the advanced age of the patients, this is a crucial situation to be considered and to be differentially diagnosed from other causes of gastric outlet obstruction, such as gastric cancer and peptic stenosis especially in elderly women [1, 7].

Laboratory studies may demonstrate leucocytosis, hydroelectrolytic and acid-base alterations as well as renal failure [1]. The grade depends on the comorbidity, the intensity of the inflammatory response and the compensatory mechanisms of the individual [1]. Less common are the alterations of the hepatic function probes and the elevation of the serum amylase [1]. Findings of obstructive jaundice with elevation of the total and direct bilirubin, ALP and *γ*GT are possible [6] depending on the anatomic level of the obstruction. 

 Abdominal plain radiography is usually of low diagnostic value because of the unspecific signs revealed [1]. In 10–50% of the cases it demonstrates the elements of the Rigler triad: bowel obstruction, pneumobilia and a calcified ectopic gallstone [1, 5–7]. The migration of a previously observed stone may also be noticed [5, 6], as well as two air fluid levels in the right upper quadrant due to the presence of air in the gallbladder [5], or dilatation of the stomach [5].

An oral contrast meal followed by a plain abdominal X-ray series could be very helpful as demonstrated in our patient, giving detailed information about the anatomic elements involved, as well as the topographic relations. As a simple and low-cost examination with high diagnostic value, we highly recommend it in cases when Bouveret syndrome is suspected, if the patient can tolerate oral contrast intake. 

 Ultrasonography may be helpful for the diagnosis but often presents a confusing picture due to the limitations determined by the subsequent anatomic alterations such as intestinal distension, collapse or presence of air in the gallbladder [1, 6, 7]. If collapsed or air-filled the gallbladder cannot be well visualised [7]. In addition when the gallbladder is contracted, it may be difficult to detect the exact location of the stone—orthotopic or ectopic [6]. The fistula may be visualised when filled with air or fluid but may be also confused with the common bile duct [6]. Ultrasonography can also reveal pneumobilia and gastric dilatation [6].

 The best imaging technique to identify the elements of the Rigler triad is CT. This is possible in 75% of cases [5]. Apart from pneumobilia, intestinal obstruction, an ectopic calculus and a dilated stomach, CT can also depict the biliodigestive fistula when the tract is enhanced by positive oral or air contrast material, or suspect its presence indirectly when the gallbladder is filled with oral contrast [1, 6]. The calculus is apparent in the duodenum in most cases [6]; however, in 15–25% of patients its visualization is not possible due to isoattenuation to bile and fluid [1, 6, 7]. Oral contrast improves the diagnostic sensitivity of CT as it surrounds the gallstone [6]. In patients with intense emesis or intolerance to oral contrast as well as in cases with isoattenuating stones the role of MRCP can be important, as it distinguishes stones from fluid, visualises the fistula with major precision and does not require the use of oral contrast material [1, 6, 7].

 The optimal treatment of patients with Bouveret syndrome still remains controversial in the world literature. The therapeutic strategy should be planned taking into consideration many parameters such as the general condition of the patient, his age and comorbidities, the location of the obstruction, the local inflammatory status, the size of the calculus and fistula [4], and the presence of more than one gallstone. The evolution can be influenced by a delay in setting the correct diagnosis and applying the proper treatment.

 The primary goal is to raise the obstruction by removing the impacted stone. This can be achieved endoscopically, surgically or by using other techniques. Modern management focuses on less invasive techniques taking into account the advanced age and serious concomitant illnesses of the majority of the affected patients [7].

 Endoscopy seems to play an important role due to its less invasive character and the lower rate of complications. However, the presence of an experienced endoscopist is usually necessary [1]. Grove, in 1976, was the first who established the diagnosis with direct endoscopic visualization of the gallstone [2]. Nowadays many endoscopic techniques have been reported and applied worldwide with various success such as endoscopic removal, net extraction, mechanical lithotripsy, electrohydraulic lithotripsy and intracorporeal laser lithotripsy, or combinations of these techniques [1, 2, 4, 6, 7]. Extracorporeal shock wave lithotripsy (ESWL) has also been performed successfully [1, 2, 4, 7]. These techniques may require more than one procedure [1]. In 1989, Holl et al. reported the first case successfully treated with ESWL [2]. The endoscopic treatment is more successful in patients with middle-sized and relatively mobile stones [1]. When the calculus is very large, endoscopy often fails [3]. Another fact that must be taken into consideration is that fragmentation of gallstones with endoscopic graspers may result in the migration and impaction of fragments at a distal part of the small bowel leading de novo to obstruction [1, 3].

 Failing endoscopic disimpaction, surgery remains the classic therapeutic method. Conventionally, Bouveret syndrome has been treated with laparotomy and enterotomy with relatively high morbidity and mortality rates of 60% and 30% respectively [7]. Open surgery, laparoscopic or laparoscopically assisted management have been reported with successful outcome in removing the impacted gallstone [5]. Endoscopically assisted minimal invasive surgery has also been described [7]. 

 During the operation the whole intestine should be examined, since in 16% of the cases other gallstones are present at another location in the digestive tract [1, 3]. If possible the stone should be removed in the stomach and extracted through a gastrotomy [1] or if this is not feasible, removal in the small bowel and extraction through an enterotomy should be attempted as performed in our patient, intending with both manoeuvres to making an incision at a healthy wall of an organ that will be healed more safely. If both manoeuvres fail, extraction with duodenotomy in the anterior surface should be performed, giving special emphasis to the optimal closure so that stenosis is avoided [1]. 

 Whether cholecystectomy and fistula repair should be performed still remains the greater controversial issue. Most authors agree that for patients in bad general condition with advanced age and serious comorbidities or in cases where the local inflammatory status makes the intervention extremely difficult predisposing to intraoperative complications, simple extraction of the obstructing gallstone is enough [2, 4]. Others, based on studies comparing combined cholecystectomy and fistula closure to enterolithotomy alone, advocate that simple extraction of the stone is adequate treatment for most patients [3, 6, 7]. Simple stone-extraction is associated with less complications and mortality rates of 12% while the respective percentage for cholecystectomy and fistula repair is 20–30% [3, 4]. In addition, a cholecystoduodenal fistula may function as a biliodigestive anastomosis if the biliary tract is permeable [4]. Another argument for the supporters of simple enterolithotomy is the fact that most patients remain asymptomatic after this procedure [2]. The recurrence of gallstone ileus is rare and since the remaining fistula is usually large, recurrent complications are rare as well [3, 5]. In cases that have not undergone cholecystectomy and fistula repair during the initial procedure, the evolution of the fistula can be variable [1]. During surveillance some fistulas get spontaneously closed within 30–60 days [1] which explains to some the further asymptomatic period in the presence of a permeable biliary tract [2], while others still remain active 90 days after initial treatment [1]. Many authors support cholecystectomy and fistula repair during the first [7] or second operation depending on the general status of the patient and the local findings [1]. An argument for that is the fact that a remaining fistula orifice favours stasis in the terminal common bile duct and therefore development of cholangitis and lithiasis [4]. Spontaneous closure of the fistula leaves a dysfunctional scleroatrophic gallbladder predisposing to recurrence of lithiasis, acute cholecystitis and gallbladder cancer [4]. Bossart et al., in 1961, have reported the development of gallbladder carcinoma in 15% of patients with biliary fistula compared with 0.8% of all gallbladder specimens at that time [4]. When the fistula orifice remains open after initial treatment, recurrent clinical manifestations such as gallbladder ileus, acute cholecystitis, cholangitis and development of cancer, occur in 5–17% of the cases [1]. Postoperative persistence of symptoms after simple calculus extraction supports the performance of cholecystectomy in patients with good general status [4]. A large retained gallstone in the gallbladder is considered by some authors as indication for cholecystectomy [6].

 In 1929, Holz was the first one who performed cholecystectomy and fistula repair after accidently penetrating through the fistula orifice in his effort to extract a gallstone from the duodenum [4].

 The technical difficulty in repairing the parietal duodenal defect may be variable and the quality of the solution given correlates to a large extent with the level of morbidity and mortality [4]. Most authors report simple suture of the fistula orifice [4]. In 1972, Redding et al. performed vagotomy and Jaboulay gastroduodenostomy in a patient with impaction of two gallstones, one in the antrum and another in the duodenal bulb, and a very extended local inflammation [4]. Others have reported closure of the fistula with a side-to-side Roux-en-Y antroduodenojejunal anastomosis in the presence of a very large and extended defect [4]. Some cases of laparoscopic treatment have also been reported [4].

 In our patient, the affected general condition prompted us to choose open approach with simple stone extraction after failure of the endoscopic techniques. During the last decade two patients with Bouveret syndrome and another 10 with gallstone ileus involving the small bowel have been treated in our department. In all cases serious comorbidity was present. All patients were treated with simple stone extraction without any intervention on the fistula. None of the patients has developed symptoms or required a second surgery due to development of associated pathology during postoperative follow-up.

 As performed in this case, we recommend the extraction of the stone through a jejunotomy if possible, when removal into the stomach is not feasible. Bouveret syndrome is a critical situation to be considered in case of gastric outlet obstruction and a diagnosis on time using the proper available means strongly affects the final outcome. An initial endoscopic effort should be generally performed. In cases requiring surgical intervention, Bouveret syndrome remains a challenge for the surgeon in terms of the right treatment. The surgical strategy should be individualised taking into consideration the patient's age, general and local status, as well as the comorbidities in correlation with the morbidity and mortality rates of each method.
